# Arsenic Content, Speciation, and Distribution in Wild *Cordyceps sinensis*

**DOI:** 10.1155/2021/6651498

**Published:** 2021-02-19

**Authors:** Yuancan Xiao, Cen Li, Wei Xu, Yuzhi Du, Ming Zhang, Hongxia Yang, Lixin Wei, Hongtao Bi

**Affiliations:** ^1^Qinghai Provincial Key Laboratory of Tibetan Medicine Pharmacology and Safety Evaluation, Northwest Institute of Plateau Biology, Chinese Academy of Sciences, Xining 810008, China; ^2^Key Laboratory of Tibetan Medicine Research, Chinese Academy of Sciences, Xining 810008, China; ^3^University of Chinese Academy of Sciences, Beijing 10049, China; ^4^Beijing Synchrotron Radiation Facility, Institute of High Energy Physics, Chinese Academy of Sciences, Beijing 100049, China

## Abstract

The excessive arsenic content in wild *Cordyceps sinensis* has caused great concerns on human health. The toxicity of arsenic depends on its concentration, chemical form, and valence. The source studies of arsenic in *C. sinensis* are essential for safety evolution and quality control. We used ICP-MS and HPLC-ICP-MS methods to determine the total arsenic amount and the arsenic speciation. Synchrotron-based XANES and micro-XRF imaging techniques were used to characterize arsenic valence and distribution. The total arsenic amount range in wild *C. sinensis* samples was 5.77–13.20 *μ*g/g with an average of 8.85 ± 2.5 *μ*g/g. As(III) and As(V) were the main species in wild *C. sinensis* samples. The iAs only accounts for 4.47–11.42% of the extracted arsenic. Trivalent and pentavalent forms were the dominant chemical forms of arsenic. Besides, we found that arsenic was accumulated at the digestive tract of the host larva.

## 1. Introduction

*Cordyceps sinensis* (*C. sinensis*) is a traditional medicine in China and eastern Asia. It is a complex of *C. sinensis* fungus and host larva that grow well at altitudes 3000–5000 m [1, 2]. *C. sinensis* occurs in the Qinghai-Tibet plateau and surrounding areas, including Tibet, Qinghai, Sichuan, Gansu, and Yunnan provinces [3]. Yushu and Guoluo prefecture of Qinghai province and Naqu region of Tibet are the core natural distribution areas [4, 5]. *C. sinensis* medicinal benefits include lung protection and kidney improvement [1, 2], immunomodulation [2, 6], antitumour effects [7], hepatoprotection [8], and increased endurance [9].

Some wild *C. sinensis* samples have a high arsenic content [10, 11] and some samples do not comply with the limit standard of BS ISO 18664:2015 [12]. Arsenic is a common element in air, soil, and groundwater [13]. Acute and chronic human exposure to arsenic causes a series of adverse health effects. These include pulmonary and respiratory, cardiovascular and hematological, gastrointestinal, hepatic, renal, neurological, immunologic, developmental, and reproduction diseases and cancer [14–16]. Arsenic is a harmful element, and most countries have established arsenic limits for drinking water, food, and medicine. The pharmacopoeia of China [1] requires the total arsenic content for most Chinese traditional medicines to be lower than 2 mg kg^−1^.

The toxicity of arsenic depends upon its concentration, chemical form, and valence. The chemical speciation of arsenic greatly affects its toxicity. Different arsenic speciations vary in their toxicity to humans. Inorganic arsenic, including arsenate and arsenite, is more toxic than organic forms like arsenosugars and arsenolipids [16]. The valence of arsenic affects its toxicity, and trivalent arsenicals are more toxic than the pentavalent forms for inorganic arsenic (iAs), monomethylarsonic acid (MMA), and dimethylarsinic acid (DMA) in vitro [16, 17]. Naranmandura [18] calculated LC50 values for human cells as 571, 843, 5.49, and 2.16 *μ*M for iAs^V^, DMA^V^, iAs^III^, and DMA^III^.

Arsenic in *C. sinensis* remains a serious problem for users. Questions to be addressed are the following: (1) What is the range of the total arsenic concentration in wild *C. sinensis*? (2) What are the chemical speciation and valence of arsenic in wild *C. sinensis* (3) What is the arsenic distribution in *C. sinensis* and where does the arsenic originate?

The total arsenic of *C. sinensis* samples has been documented [11, 19–22], and some studies have reported the arsenic speciation analysis of wild *C. sinensis* [21–25]. However, valence analysis and in vivo arsenic distribution in wild *C. sinensis* are rare. Basic research on the content, form, and valence of arsenic in *C. sinensis* is critical for understanding *C. sinensis*. The arsenic distribution in medicinal *C. sinensis* is important for arsenic source investigation. The arsenic source can be inferred from the distribution results of arsenic, and it provides data related to the accumulation and mechanism of arsenic in *C. sinensis*. Therefore, it is important and necessary to study the distribution of arsenic in *C. sinensis*.

Inductively coupled plasma-mass spectrometry (ICP-MS) [26] is a powerful technique for element detection with very low detection limits and wide linear dynamic range. ICP-MS hyphenated with chromatographic separation techniques such as liquid chromatography, gas chromatography, and capillary electrophoresis was used in elemental speciation analysis in clinical, environmental, food, and life sciences [26–28]. ICP-MS and HPLC-ICP-MS methods are useful tools for arsenic content determination and arsenic chemical species studies [29] and are applied in plant hyperaccumulation studies [30] and environmental and health studies [31].

Synchrotron-based X-ray techniques, including X-ray absorption near-edge structure spectroscopy (XANES) and micro X-ray fluorescence spectroscopy (*μ*-XRF), can provide molecular-level information and spatial imaging capabilities [32]. Synchrotron-based X-ray techniques are widely used in physics, chemistry, earth and environmental sciences, and life and agricultural sciences [32–36].

The aim of this research was to acquire information of the total arsenic content, arsenic speciation, arsenic valence, and distribution of arsenic in wild *C. sinensis* by ICP-MS, HPLC-ICP-MS, SXANS, and *μ*-XRF techniques. The data reveal the chemical form and distribution of arsenic in wild *C. sinensis*, suggest the sources of arsenic, and provide a reference for additional safety assessments of wild *C. sinensis*.

## 2. Materials and Methods

### 2.1. Chemicals and Reagents

Element reference standard solutions of arsenic (As, GBW(E)080117, 1000 *μ*g/ml), arsenite (As(III), GBW08666, 75.7 ± 1.2 *μ*g/g), arsenate (As(V), GBW08667, 17.5 ± 0.4 *μ*g/g), monomethylarsonic acid (MMA, GBW08668, 25.1 ± 0.8 *μ*g/g), dimethylarsinic acid (DMA, GBW08669, 52.9 ± 1.8 *μ*g/g), arsenobetaine (AsB, GBW08670, 38.8 ± 1.1 *μ*g/g), arsenocholine (AsC, GBW08671, 28.0 ± 1.1 *μ*g/g), and standard reference material GBW09588 (*Atractylodes macrocephala*) were obtained from the National Institute of Metrology (Beijing, China), with their concentrations guaranteed. Working mixture standard solutions of arsenic speciation were prepared daily by diluting the source standard solutions to proper concentrations with pure water. Tune solution and mixed internal standard were provided by PerkinElmer (MA, USA). Na_3_AsO_4_, NaAsO_2_, and As (Cys)_3_ were also provided by the National Institute of Metrology (Beijing, China).

Nitric acid (HNO_3_, 68%, ultrapure) and hydrogen peroxide (H_2_O_2_, 30%, ultrapure) were purchased from Suzhou Crystal Clear Chemical Co., Ltd. (Suzhou, China). High purity argon (Ar, 99.999 %) was obtained from Jinxin Gas Co., Ltd. (Xining, China). HPLC grade methanol (Merck, Germany), HPLC grade ammonium dihydrogen phosphate, and aqueous ammonia were obtained from ANPEL Laboratory Inc. (Shanghai, China). Ultrapure water (18.2 MΩ) prepared with a Milli-Q system (Millipore, Co., USA) was used for all solution preparations. The glassware and plasticware used in this experiment were soaked in 20% HNO_3_ solution for 24 h prior to use.

### 2.2. Sample Collection and Preparation

Six wild *C. sinensis* samples (C1–C6) (each 20 g) were purchased from native habitats, Zaduo, Chengdu, Yushu, Nangqian, Zhiduo, and Qumalai county, Yushu prefecture, Qinghai province, China. Samples were authenticated by professor Yuzhi Du, a certified pharmacist of traditional Chinese medicine.

Samples were rinsed with deionized water to remove dust and soil from surface, dried at 40°C for 48 h, and then stored at −20°C before use. Before experiments, the samples were ground into powder and passed through a 40 mesh sieve.

### 2.3. Instrumentation

A NexION^™^ 350D ICP-MS (PerkinElmer, Waltham, MA, USA) and A-30 UPLC (PerkinElmer, Waltham, MA, USA) were used. The separation of arsenic species was performed on a PerkinElmer Altus A-30 UPLC system, equipped with a solvent delivery module (quaternary pump) and sampling module. Separation was achieved using a Hamilton PRP100 column (250 mm × 4.6 mm id, 5 *μ*m) (Hamilton, Sweden). The ICP-MS was operated on the standard mode. The pH values were measured using a Mettler Toledo FiveEasy Plus pH meter (Mettler Toledo Co., Shanghai, China). Milli-Q purified water was obtained from a Milli-Q (reference) purified water apparatus (Millipore Co., USA). A microwave oven (MASTER 40 Digestion/Extraction/Synthesis Microwave Labstation) equipped with forty 70 ml TFM Teflon vessels, with an energy output of 3600W, was used to digest samples. The maximum digestion temperature and pressure were 220°C and 3 Mpa, respectively. An ECH-20 digital temperature control heater was used for evaporating excess nitric acid. The microwave oven and ECH-20 digital temperature control heater were products of Sineo Microwave Chemistry Technology Co., Ltd. (Shanghai, China).

The XAFS experiment end station 1W1B, Beijing Synchrotron Radiation Facility (Beijing, China); micro X-ray fluorescence beamline BL15U at Shanghai Synchrotron Radiation; infrared tablet presser (HY-12, Tianjin Skylight Optical Instrument Co., Ltd); vacuum freeze dryer (FD-1D-50, Beijing Boyikang Laboratory Instrument Co., Ltd); frozen microtome (CM 1950, Leica Co., Ltd, Germany); and positive optical microscope imaging system (E200, Sony Co., Ltd., Japan) were used.

### 2.4. Determination of Total Arsenic

#### 2.4.1. Sample Ingestion

An ICP-MS system coupled with the microwave digestion technique was used for sample preparation and detection. *C. sinensis* powder, 0.25 g, was decomposed using microwave equipment with a mixture of HNO_3_ (4.0 mL) and H_2_O_2_ (2.0 mL). The operating program of the microwave system was as follows: the samples were heated to 120°C from room temperature in 5 min and held for 5 min, then heated to 160°C in 5 min and held for 10 min, and heated to 200°C in 15 min and held for 15 min. During the digestion process the wave power was set to 1800 W. After digestion, the samples were cooled to room temperature. Excess HNO_3_ was removed by heating the sample solution at 120°C for 20 min. The digestion sample solutions were cooled to room temperature and diluted with ultrapure water up to 50 mL.

#### 2.4.2. Conditions and Methods

The analysis conditions, including RF power, plasma gas flow, auxiliary gas flow, nebulizer gas flow, sampling depth, and peristaltic pump rate, were 1250 W, 18 L/min, 1.2 L/min, 0.72 L/min, 6 mm, and 35 r/min, respectively. Besides, selected isotope m/z 75 was detected ion. Samples were quantified with external calibration curve As standards (calibration points: 1, 5, 10, 20, and 50 ng/mL), and internal standards (40 ng/mL of ^72^Ge) were used for metal determination by ICP-MS. Before determination, the status of ICP-MS was adjusted to optimum with the tuning solution. The internal standard was used, and internal standard solution was introduced into the sample flow with a *T* shape pipe online. Triplicate analyses were performed for each sample. The corresponding digestion blanks (reagent blanks) were also measured. The arsenic of CRM GBW09588 (*Atractylodes macrocephala*) was determined and used for quality control purposes using the same methods.

### 2.5. Arsenic Speciation Analysis

#### 2.5.1. Sample Preparation

The extraction methods were performed with reference to the method of Guo et al. [21] and Zhou et al. [23]. Approximately 0.5 g of each powder sample of *C. sinensis* was added to 10 mL of 0.15 mol/L dilute nitric acid solution and soaked in the mixture overnight. Then, the mixtures were heated in an incubator for 150 min at 90°C and shaken for 1 min every 30 min. The mixtures were cooled to room temperature and centrifuged at 8000 r/min after heat extraction. The supernatants were removed, and 5 mL of 0.15 mol/L dilute nitric acid solution was added to the residue. The extraction was repeated using the procedure described above. The combined supernatants from the two rounds of extraction were analyzed immediately after filtration with a 0.22 *μ*m PTEF membrane. A corresponding reagent blank was made, and the procedure was performed in triplicate.

#### 2.5.2. Arsenic Speciation Analysis Method

The different species of arsenic were separated by HPLC and detected by ICP-MS. An anion-exchange column (Hamilton RPR 100 column, 250 mm × 4.6 mm, 5 *μ*m) was used for separation with a gradient system of eluent A, 10 mmol/L NH_4_H_2_PO_4_ (containing 1% methanol V/V, NH_3_·H_2_O adjusted pH 9.7), and eluted with B, 40 mmol/L NH_4_H_2_PO_4_ (containing 1% methanol V/V, NH_3_·H_2_O adjusted pH 6.7) solution, at a flow rate of 1.0 mL/min. The HPLC elution condition was achieved using the following procedures. For eluent A, 100.0% initial proportion, 100.0% maintained for 4.0 min; linear decrease to 0.0% at 4.5 min and 0.0% maintained for 13.5 min; linear increase to 100.0% at 14.0 min; equilibrium maintained for for 4.0 min. The total sample injection time was 18.0 min, and the acquisition time was 14 min. The injection volume was 10.0 *μ*L.

The method of arsenic speciation analysis was validated by the standard addition method, and the recoveries of each arsenic speciation were used for evaluating the method feasibility. The total arsenic in the extraction solution and the residue of the extracted sample were ingested by microwave digestion method, and the total arsenic was determined by the method in Section 2.4. Then, total arsenic results of extraction solution and residue of extraction were compared with the results of sum of six arsenic species in *C. sinensis*.

### 2.6. Arsenic Valance Analysis In Vivo

#### 2.6.1. Sample Preparation and Reference Materials

*C. sinensis* samples were ground into fine powder and then pressed into round tablet with 1 cm diameter. All samples were coded and examined by X-ray absorption spectroscopy. The reference materials Na_3_AsO_4_ and As (Cys)_3_ were used.

### 2.7. Arsenic Distribution in Wild *C. sinensis*

#### 2.7.1. Sample Preparation

**Figure 1:**
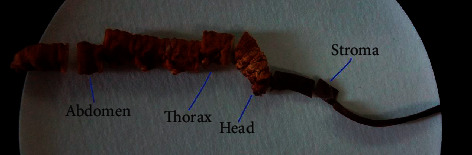
The location of thin slice on *C. sinensis*.

A *C. sinensis* sample from Zhiduo county was frozen, and sections of 50 *μ*m thickness were made from the stroma, head, thorax, and abdomen (the location of the thin slices of the samples is shown in Figure 1) and pasted on the XRF tape (TF-500) for *μ*-XRF imaging.

#### 2.7.2. Facility Conditions

The distribution of As in *C. sinensis* samples was analyzed with *μ*-XRF at the beamline BL15U at the Shanghai Synchrotron Radiation Facility (SSRF, Shanghai, China). The continuous synchrotron X-rags were monochromatized by a Si(111) double-crystal monochromator. A monochromatic X-ray beam with photon energy of 13 keV was used to excite the samples. The cross section of the beam irradiation on the samples was adjusted to about 200 × 200 mm^2^ with an about 10^11^ phs/s photon flux. The sample was placed at a 45° angle to the incident X-ray beam, and X-ray fluorescence was detected with a 50 mm^2^ silicon drift detector (Vortex, USA) oriented at a 90° angle to the incident beam. A light microscope was coupled to a computer for sample viewing. The sample platform was moved by a motorized x-y mapping stage. The As distributions in the sections of different parts of the caterpillar were continuously scanned at a step of 125 *μ*m for both *x* and *y* directions. Each spot was irradiated for 5 s.

#### 2.7.3. Data Analysis

The X-ray spectra were analyzed by the AXIL program (Canberra Benelux, Belgium), and all the element fluorescence intensities and the Compton scattering intensity were normalized to the collecting time and the changes in I0, which was measured by an upstream ion chamber. The relative quantitative images of metals were obtained using software IGOR Pro 6 (WaveMetrics Inc., USA).

## 3. Results

### 3.1. Total Arsenic Content of Wild *C. sinensis* Samples

The amount of total arsenic in wild *C. sinensis* was determined by microwave digestion coupled with the ICP-MS method. Total arsenic content of the six *C. sinensis* samples ranged from 5.77 to 13.20 *μ*g/g, with RSD of 2.1–5.4% and mean of 8.85 ± 2.5 *μ*g/g (details are provided in Table 1 and Supplementary Table 1).

The ^75^As standard curve was *Y* = 0.010*X* + 0.002 with a correlation coefficient (*r*) of 0.9999, and the detection limit was 0.011 ng/ml. The total As of CRM GBW09588 was 0.202 ± 0.005 *μ*g/g (*n* = 3). This result was similar to the certificate value of 0.211 ± 0.008 *μ*g/g.

### 3.2. Arsenic Speciation of Wild *C. sinensis*

**Figure 2:**
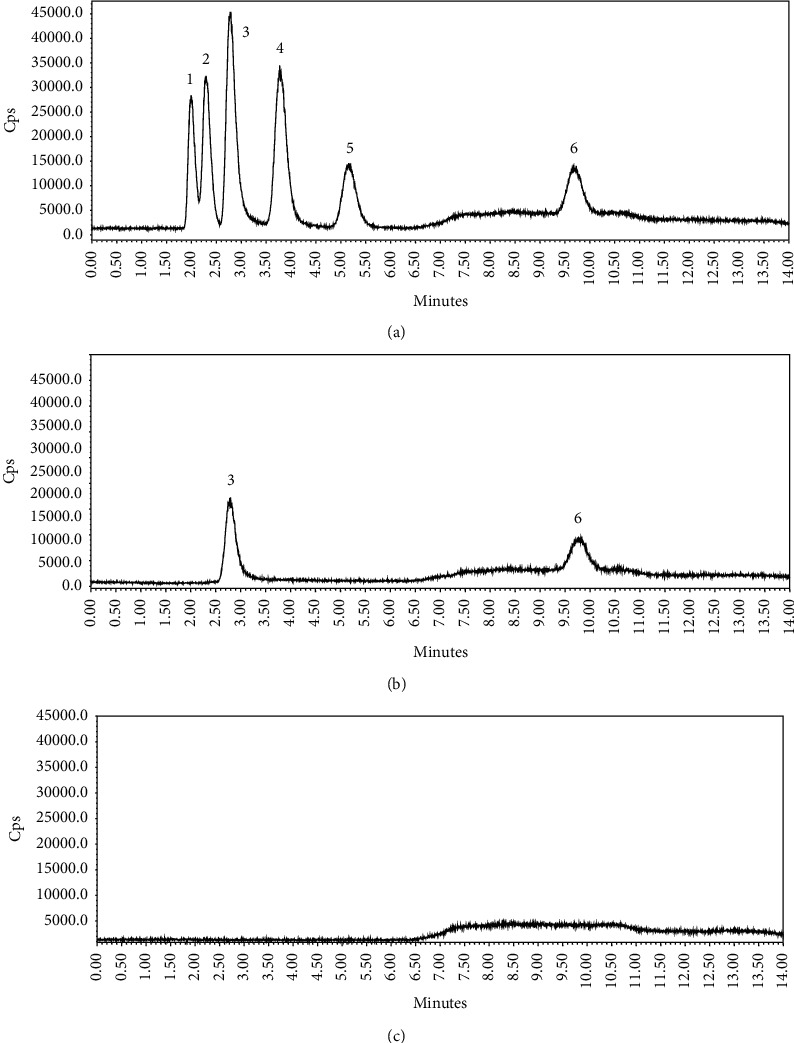
Chromatogram of arsenic speciation analysis of wild *C. sinensis* using the HPLC-ICP-MS method: (a) mixed standard of six arsenic species; (b) sample of wild *C. sinensis*; (c) blank (0.15 mol/L HNO_3_). 1: AsC; 2: AsB; 3: As(III); 4: DMA; 5: MMA; 6: As(V).

**Figure 3:**
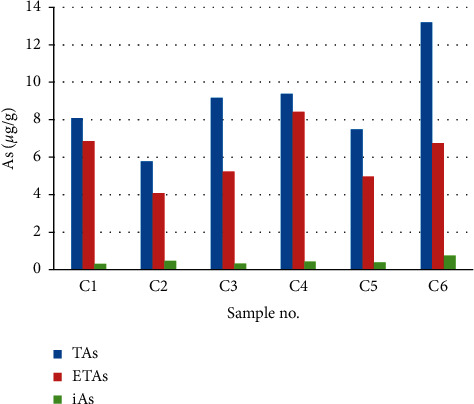
Content of TAs, ETAs, and iAs in wild *C. sinensis*. TAs represent total arsenic of *C. sinensis*, ETAs represent total extracted arsenic with 0.15 mol/L HNO_3_ of *C. sinensis*, and iAs represents the sum of As(III) and As(V) of extracted solution.

The arsenic speciation analysis was performed on a HPLC-ICP-MS using the established method. The representative chromatograms for typical separation of arsenic species are shown in Figure 2. The analysis methodology data including regression equations, correlation coefficients, linear ranges, detection limits, and recoveries are shown in Table 2. The recoveries of As(III), As(V), MMA, DMA, AsC, and AsB were 94.4%, 79.6%, 95.6%, 96.7%, 93.7%, and 94.9%, respectively. Accordingly, we can state that the results of recovery of HPLC-ICP-MS method are reliable. The contents of different arsenic species in the samples are shown in Table 3 and Figure 3.

### 3.3. Arsenic Valence of Wild *C. sinensis*

**Figure 4:**
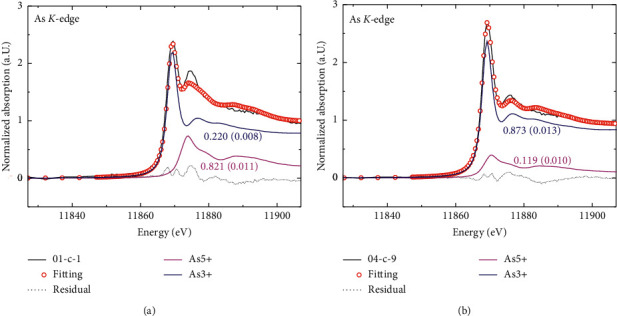
Experimental and fitted spectra of two samples (C1 and C4).

The raw XAFS data of *C. sinensis* samples and the references were preprocessed through conventional procedures by normalizing to the unit edge jump after removing the atomic background as implemented in the IFEFFIT package, shown in Figure 4. The Fourier transforms of k2-weighted EXAFS were conducted over the *k* range [3–10 Å^−1^] for all samples. Speciation analysis was conducted on the XANES region, the 20 eV below and 50 eV above the absorption edge, for all samples using the selected standards. Due to the limitation of the standards library, the useful standards were selected by comparing the spectral fingerprint of the sample and that of the standards. We narrowed the fingerprint to two compounds, i.e., Na_3_AsO_4_-As^5+^ and As-Cys-As^3+^, each of which showed distinctive fingerprints in the XANES region (details are provided in Supplementary Figure 1).

### 3.4. Distribution of Arsenic in Wild *C. sinensis*

**Figure 5:**
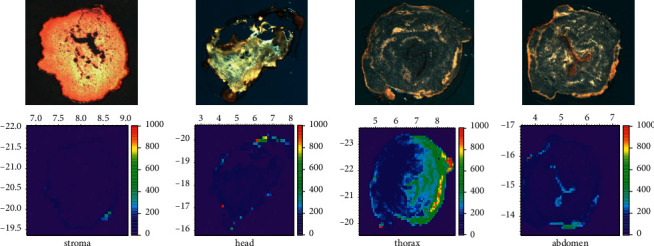
Arsenic distribution in wild *C. sinensis*.

The different *C. sinensis* sections of stroma, larva head, larva thorax, and larva abdomen were imaged using the synchrotron radiation *μ*-XRF method. Sampling location is shown in Figure 1, and As distribution is shown in Figure 5. The relative level of arsenic is represented by the intensity or counts of fluorescence photons. Figure 5 shows the As micro distribution in the host body of *C. sinensis.* The micro distribution of As shows that As exists in the larva and is concentrated in the mid-thorax and the abdomen. We speculated that it was the digestive tract according to the location and shape of As focusing. Compared to the larval thorax and abdomen, the stroma and head had low photon counts, indicating their low As content.

## 4. Discussion

### 4.1. Total Arsenic Content of Wild *C. sinensis*

The total As results in this study were consistent with previous studies of wild *C. sinensis.* The total arsenic results of Guo [21] were 4.00–5.25 mg/kg in *C. sinensis* samples from Litang, Naqu, and Yushu. They were 2.560–5.590 mg/kg with mean of 1.032 ± 0.989 mg/kg in 45 samples from a Beijing market that were collected from major origins of wild *C. sinensis* in Lu's studies [19]. Li [20] documented 5.9–12.5 mg/kg of five samples from Qinghai, Tibet, and Gansu provinces. Zuo et al. [22] found 8.53 ± 3.49 mg/kg in 34 samples from Qinghai, Tibet, Sichuan, Gansu, and Yunnan provinces. Zhou et al. [23] found 9.70 ± 0.62 mg/kg in a sample from Qinghai province.

### 4.2. Arsenic Speciation Concentration of Wild *C. sinensis*

For six arsenic species, AsC, AsB, As(III), MMA, DMA, and As(V), were separated well in 14 min (Figure 2). Performance parameters of established methods (Table 2) showed that the detection limits for the six arsenic species ranged from 0.41 *μ*g/L to 2.06 *μ*g/L. The standard curve prepared for each arsenic species was linear, and the correlation coefficients were 0.9991 to 0.9999. The relative standard deviations (RSDs %) were less than 5%.

The arsenic speciation results showed that AsC, AsB, MMA, and DMA were not detected in the six samples of wild *C. sinensis* from Yushu prefecture. Two inorganic arsenic species, As(III) and As(V), were the major species in wild *C. sinensis*. Their concentration ranged from 0.126 ± 0.002 *μ*g/g to 0.473 ± 0.058 *μ*g/g, and 0.180 ± 0.006 *μ*g/g to 0.265 ± 0.016 *μ*g/g, respectively. The total iAs was 0.307–0.738 *μ*g/g and amounted to 4.47–11.42% of the total arsenic. Compared with the total arsenic result of *C. sinensis* (Table 1), the extracted arsenic was less than the former.

The total arsenic results of extraction solution and residue of extraction were compared with the results of the total arsenic of *C. sinensis* samples by microwave digestion method (Tables 1 and 3). The sum of total arsenic in the extraction and residue is basically consistent with the total arsenic content of each *C. sinensis* sample.

Using the same extraction solution of 0.15 mol/L HNO_3_ and temperature of 90°C, the arsenic speciation results of the present study were in partial agreement with the results of Guo et al. [21] who discovered AsB in *C. sinensis* samples from Litang, Naqu, and Yushu, and the iAs content ranged from 0.31 to 0.38 *μ*g/g (6.0– 8.3%). The extraction time for these two studied samples differed, with 12 hours for Guo et al. [21] and 2.5 hours for the present study. Guo et al. [21] had an extraction efficiency of 92.3–104% for total arsenic, which is higher than that in the present study (51.1–89.7%).

In arsenic speciation analysis, different extraction methods produce different results. HNO_3_ solution is superior for recovery of iAs compared to simulated gastrointestinal juice. The results of Guo et al. [21], Zuo et al. [22], Zhou et al. [23], Li et al. [24], and the present research all support this conclusion.

Unknown arsenic species exist in wild *C. sinensis* based on the present study and published results [22–24, 37]. Other studies indicate that the unknown arsenic species were in organic form and Guo's results [22] supported this conclusion using an H_2_O_2_ oxidation test. Li et al. [24] showed that unknown organic arsenic was abundant in wild *C. sinensis* using SEC-HPLC- ICP- MS and continuous extraction methods. Arsenic is distributed in lipids, proteins, polysaccharides, and other chemical compositions [24].

### 4.3. Arsenic Valence

The speciation of the samples was analyzed using linear combination fitting (LCF), which is implemented in the ATHENA-IFEFFIT package. By carefully calibrating the energy and allowing a small shift of energy as a fitting parameter, the proportion of constituent species can be obtained by linear combinations of the selected standards. The acceptable criterion for LCF is to obtain fitting with minimum residual and full physical meaning, shown in Figure 4.

The LCF was conducted for the XANES region: –20 eV to + 60 eV from the edge. We did not constrain the sum of the individual weights, because there should be some missing references that are not included in the standards library. Fortunately, the major constituents are included in the analysis. There are spectral features that cannot be fully reproduced, considering the difference between the natural/complex samples and the synthesized/simpler standards.

### 4.4. Arsenic Distribution and Source of Arsenic

Arsenic was concentrated in the larva digestive tract, which indicates that the arsenic source is the larval food. This is likely because the host larva spends most of its life in the soil, and soil humus is documented as a larval food. The results of stable isotope composition showed that [38] soil humus is one of the foods for the host of *C. sinensis* in the Qinghai-Tibet plateau. Additionally, the levels of As in *Cordyceps* are correlated with the soil samples from their collection locations [39], suggesting that As in *C. sinensis* likely originates from the soil.

## 5. Conclusion

The study documented high total arsenic concentrations in wild *C. sinensis* with most levels exceeding 2 mg/kg. Most arsenic detected was inorganic, and it existed in trivalent and pentavalent forms. The soil and food of the host larva of caterpillar of *C. sinensis* were the source of arsenic. Unknown organic arsenic species exist in a large amount in *C. sinensis*; therefore, the chemical properties and toxicity of the unknown organic arsenic speciation decide the health risk of *C. sinensis*.

## Figures and Tables

**Table 1:** Total arsenic content of wild *C. sinensis*.

No.	Sample	Origin	As (mg/kg)	RSD (%) (*n* = 3)
1	C1	Yushu city	8.08	2.8
2	C2	Nangqian county	5.77	2.2
3	C3	Qumalai county	9.18	2.9
4	C4	Chengdu county	9.38	4.4
5	C5	Zhiduo county	7.49	2.1
6	C6	Zaduo county	13.20	5.4
Average			8.85	
SD			2.50	

**Table 2:** Regression equations, correlation coefficients, linear ranges, detection limits, and recoveries of HPLC-ICP-MS method.

Analyte	Linear equation	*R*	Linear range (*μ*g/L)	LOD (*μ*g/L)	Recovery (%)
AsC	*Y* = 11589X − 10045	0.9999	4.13–264.01	0.41	93.7
AsB	*Y* = 12052*X* + 19812	0.9998	5.05–322.95	1.35	94.9
As(III)	*Y* = 9814.4*X* + 28579	0.9997	10.77–689.02	0.35	94.4
DMA	*Y* = 12006*X* + 34041	0.9996	7.98–510.86	0.80	96.7
MMA	*Y* = 10794*X* + 13051	0.9993	3.76–240.38	1.51	95.6
As(V)	*Y* = 12410*X* − 15367	0.9991	2.75–176.27	2.06	79.6

**Table 3:** As speciation analysis in wild *C. sinensis* preparations by 0.15 mol/L HNO_3_.

No.	AsC	AsB	As(III)^a^	DMA	MMA	As(V)^a^	iAs	ETAs^b^	RTAs^c^	iAs^d^ (%)
C1	ND	ND	0.127 ± 0.007	ND	ND	0.180 ± 0.006	0.307	6.858	1.103	4.47
C2	ND	ND	0.202 ± 0.004	ND	ND	0.263 ± 0.017	0.465	4.073	1.546	11.42
C3	ND	ND	0.126 ± 0.002	ND	ND	0.191 ± 0.013	0.316	5.231	3.722	6.05
C4	ND	ND	0.215 ± 0.001	ND	ND	0.208 ± 0.025	0.422	8.415	0.917	5.02
C5	ND	ND	0.194 ± 0.043	ND	ND	0.196 ± 0.000	0.391	4.949	2.435	7.89
C6	ND	ND	0.473 ± 0.058	ND	ND	0.265 ± 0.016	0.738	6.745	5.984	10.94

*Note.* ND: not detected. ^a^Data are the mean ± SD, *μ*g/g. ^b^Total arsenic of sample extracted solution, *μ*g/g. ^c^Arsenic content of residues after the extraction, *μ*g/g. ^d^Percentage of inorganic arsenic in extracted solution (%).

## Data Availability

The data are included within the article.

## Supplementary Materials

Supplementary Materials Supplementary Figure 1: XAFS data of six *Cordyceps sinensis* samples. Supplementary Table 1: total arsenic content of wild *Cordyceps sinensis* samples.
