# Supplementary material for: Arsenic Content, Speciation, and Distribution in Wild Cordyceps sinensis
Source: Evid Based Complement Alternat Med. 2021 Feb 19;2021:6651498. doi: 10.1155/2021/6651498 (PMC7910042; doi:10.1155/2021/6651498)
Supplement: Supplementary Materials — Supplementary Figure 1: XAFS data of six Cordyceps sinensis samples. Supplementary Table 1: total arsenic content of wild Cordyceps sinensis samples. [file 6651498.f1.docx]

**Supplementary Materials Lists**

***Supplementary Figure 1*: XAFS Data of Six *Cordyceps sinensis* Samples**

***Supplementary Table 1：Total arsenic content of wild Cordyceps sinenesis samples***

***Figure 1*: XAFS Data of Six *Cordyceps sinensis* Samples**

(Note: C1 (01-c-1) is the sample of Yushu county; C2 (02-c-3) is the sample of Nangqian county; C3 ( 03-c-7) is the sample of Qumalai county; C4 (04-c-9) is the sample of Chengduo county; C5 (05-c-11) is the sample of Zhiduo county; C6 (06-c-16) is the sample of Zaduo county; the county of Yushu, Nangqian, Qumalai, Chengduo, Zhiduo and Zaduo are the counties of Yushu prefecture, Qinghai province, China) .

***Table 1：Total arsenic content of wild Cordyceps sinenesis samples***

|  | NO. | Weight(g) | Volume(mL) | As (ng/mL) | As（μg/g) | Average(μg/g) | STDEV | RSD(%) |
| --- | --- | --- | --- | --- | --- | --- | --- | --- |
| Blank | ACK-1 |  | 50 | 0.318 | 0.406 |  |  |  |
|  | ACK-2 |  | 50 | 0.493 |  |  |  |  |
| C1 | C1-1 | 0.1937 | 50 | 31.655 | 8.066 | 8.08 | 0.227 | 2.8 |
|  | C1-2 | 0.2009 | 50 | 33.838 | 8.321 |  |  |  |
|  | C1-3 | 0.2026 | 50 | 32.284 | 7.867 |  |  |  |
| C2 | C2-1 | 0.1995 | 50 | 23.797 | 5.863 | 5.77 | 0.126 | 2.2 |
|  | C2-2 | 0.204 | 50 | 24.148 | 5.819 |  |  |  |
|  | C2-3 | 0.2017 | 50 | 23.100 | 5.626 |  |  |  |
| C3 | C3-1 | 0.2034 | 50 | 37.145 | 9.031 | 9.18 | 0.268 | 2.9 |
|  | C3-2 | 0.2012 | 50 | 38.581 | 9.487 |  |  |  |
|  | C3-3 | 0.2021 | 50 | 36.840 | 9.014 |  |  |  |
| C4 | C4-1 | 0.2016 | 50 | 36.366 | 8.919 | 9.38 | 0.409 | 4.4 |
|  | C4-2 | 0.2021 | 50 | 39.581 | 9.692 |  |  |  |
|  | C4-3 | 0.203 | 50 | 39.129 | 9.538 |  |  |  |
| C5 | C5-1 | 0.2001 | 50 | 31.102 | 7.670 | 7.49 | 0.155 | 2.1 |
|  | C5-2 | 0.2042 | 50 | 30.540 | 7.378 |  |  |  |
|  | C5-3 | 0.2008 | 50 | 30.256 | 7.433 |  |  |  |
| C6 | C6-1 | 0.2037 | 50 | 56.369 | 13.737 | 13.20 | 0.709 | 5.4 |
|  | C6-2 | 0.2019 | 50 | 54.809 | 13.473 |  |  |  |
|  | C6-3 | 0.2015 | 50 | 50.368 | 12.398 |  |  |  |

(Note: C1 is the sample of Yushu county; C2 is the sample of Nangqian county; C3 is the sample of Qumalai county; C4 is the sample of Chengduo county; C5 is the sample of Zhiduo county; C6 is the sample of Zaduo county; the county of Yushu, Nangqian, Qumalai, Chengduo, Zhiduo and Zaduo are the counties of Yushu prefecture, Qinghai province, China) .
